# Early Diagnosis of Alzheimer’s Disease in Human Participants Using EEGConformer and Attention-Based LSTM During the Short Question Task

**DOI:** 10.3390/diagnostics15040448

**Published:** 2025-02-12

**Authors:** Seul-Kee Kim, Jung Bin Kim, Hayom Kim, Laehyun Kim, Sang Hee Kim

**Affiliations:** 1 Bionics Research Center, Korea Institute of Science and Technology, Seoul 02792, Republic of Korea; seulki120@kist.re.kr; 2 Department of Brain and Cognitive Engineering, Korea University, Seoul 02841, Republic of Korea; 3 Department of Neurology, Korea University Anam Hospital, Korea University College of Medicine, Seoul 02841, Republic of Korea; kjbin80@korea.ac.kr (J.B.K.);; 4 Department of HY-KIST Bio-Convergence, Hanyang University, Seoul 04763, Republic of Korea

**Keywords:** Alzheimer’s disease, subjective cognitive decline, cognitive task, EEG, deep learning, mild cognitive impairment, neurodegenerative biomarkers

## Abstract

**Background/Objectives**: Alzheimer’s disease (AD) is a progressive neurodegenerative disorder advancing through subjective cognitive decline (SCD), mild cognitive impairment (MCI), and dementia, making early diagnosis crucial. Electroencephalography (EEG) is a non-invasive, cost-effective alternative to advanced neuroimaging for detecting early neural changes. While most studies focus on resting-state EEG or handcrafted features with traditional machine learning, deep learning (DL) offers a promising tool for automated EEG analysis. This study classified the AD spectrum (SCD, MCI, AD) using EEG recorded during resting-state and task-based conditions. Specifically, EEG was recorded during a simple yes/no question-answering task, mimicking everyday cognitive activities, and was explored. We hypothesized that brain activity during tasks involving listening, comprehension, and response execution provides diagnostic insights. **Methods**: We collected 1 min of resting-state EEG and approximately 3 min of task-based EEG from 20, 28, and 10 participants with SCD, MCI, and AD, respectively. Task data included response accuracy and reaction time. After minimal preprocessing, two DL models, attention long short-term memory and EEGConformer, were used for binary (e.g., SCD vs. MCI) and three-class (SCD, MCI, AD) classification. **Results**: Task-based EEG outperformed resting-state EEG, with a 5–15% improvement in accuracy. The area under the curve (AUC) results consistently demonstrated superior classification performance for task-based EEG compared to resting-state EEG across all group distinctions. No significant performance difference was observed between the two DL models. **Conclusions**: We proposed a cognitive task-based approach for early AD spectrum diagnosis via EEG, offering greater accuracy by leveraging advanced DL models.

## 1. Introduction

Alzheimer’s disease (AD) is a neurodegenerative disorder primarily affecting older adults and is the leading cause of dementia [1,2]. Pathologically, AD is marked by the accumulation of amyloid-beta (Aβ) plaques and tau protein neurofibrillary tangles in the brain, with neuronal damage and cell death preceding cognitive impairment by decades [3,4]. Clinically, AD is characterized by progressive memory loss, cognitive decline, and behavioral changes. AD is now recognized as a continuum, termed the AD spectrum, rather than distinct stages [5]. This spectrum includes subjective cognitive decline (SCD), mild cognitive impairment (MCI) attributed to AD, and dementia resulting from AD [6]. MCI serves as an intermediate stage between normal aging and pathological cognitive decline [7], while SCD describes individuals reporting subjective cognitive decline but performing normally on objective cognitive tests [8]. SCD is considered a preclinical stage of AD that may progress to MCI and, eventually, dementia. Early AD diagnosis is critical to enable timely interventions that may slow disease progression, emphasizing the importance of diagnostic methods for MCI and the earlier SCD stage [9]. While studies have extensively focused on distinguishing healthy controls (HCs) from patients with MCI and AD [9,10,11], research on differentiating SCD from MCI remains limited [12]. Advanced neuroimaging techniques, such as positron emission tomography and magnetic resonance imaging, can capture structural and functional brain changes associated with AD. However, their high cost, invasiveness, and logistical challenges limit their utility in clinical settings [13]. In contrast, electroencephalography (EEG) offers a non-invasive, portable, and cost-effective alternative that detects subtle neuronal changes before structural brain damage or behavioral symptoms emerge, making it a promising tool for early AD diagnosis [9,14,15].

Despite extensive research on EEG-based early diagnosis of AD, the complexity of brain changes in its early stages poses significant challenges for developing accurate biomarkers to diagnose and monitor AD progression [9,11]. Many studies have relied on hand-crafted EEG features combined with traditional machine learning (ML) approaches, such as support vector machines or k-nearest neighbors [9,16,17]. While these methods reflect specific neurological features, their dependence on multiple signal processing steps, including feature extraction, threshold selection, and manual tuning, makes the results highly dataset-dependent and limits generalizability. In light of these limitations, deep learning (DL) has emerged as a promising tool for EEG analysis. DL models automatically extract and learn patterns from raw EEG data, capturing hidden brain activity that manual feature extraction may overlook. Additionally, DL reduces preprocessing needs, enabling robust performance with minimally processed data. For instance, Miltiadous et al. combined a convolutional neural network (CNN) with a Transformer architecture to classify HC and AD using resting-state EEG, achieving 83.28% accuracy [15]. Alvi et al. classified HC and MCI with 96.41% accuracy using an LSTM model [18]. Similarly, Sibilano et al. developed a Transformer-based DL framework to classify HC, SCD, and MCI using resting-state EEG, achieving 76.2% accuracy for SCD vs. MCI classification and 54.2% for three-class classification [9]. Although these studies demonstrated good performance in detecting AD using deep learning models, many reported validation results rather than employing separate test data for model evaluation. Kim et al. utilized a large-scale resting-state EEG dataset to train the model and reported an accuracy of 74.66% for the three-class classification of HC, MCI, and AD [17]. Similarly, Kumar [19] and Imani [20] optimized LSTM-based models, achieving 96.17% and 93.00% accuracy for classifying HC and AD, respectively. Their studies improved reliability by using a separate test set for evaluation rather than relying solely on validation data. Notably, their methodology included subject-wise validation, ensuring a strict separation of training and test datasets and addressing overfitting issues associated with trial-by-trial validation [21]. This rigorous approach further enhanced the reliability of their findings. However, these studies involved lengthy data acquisition processes, requiring resting-state EEG recordings of at least 5 min and up to 30 min. While classification performance for HC and AD was high, these studies did not address classification between SCD, MCI, and AD within the AD spectrum.

Most studies diagnosing AD with EEG have focused on resting-state data. However, AD is fundamentally a disorder of cognitive decline, suggesting that brain activity during cognitive tasks may provide more direct insights into the disease. Given EEG’s high temporal resolution, it is well suited for task-based studies, prompting growing interest in using task-based EEG to diagnose AD. For example, Ho et al. employed N-back and Oddball tasks to classify HC and AD, achieving accuracies of 73.70% and 75.95%, respectively, using a CNN-LSTM hybrid model [22]. Similarly, Crook et al. recruited young adults, older adults, and patients with MCI to perform a working memory task and two prospective memory tasks, achieving classification accuracies of 73.94% (working memory task), 83.33% (perceptual prospective memory task), and 80% (conceptual prospective memory task) using a spiking neural network model [23]. These findings demonstrate that brain activity varies depending on the cognitive task performed, highlighting the potential of task-specific EEG for AD diagnosis. However, existing cognitive tasks, such as the N-back or Go/No-go task, require hundreds of repeated trials to reduce EEG noise and clarify ERP patterns. This can be challenging for older adults, particularly those with cognitive decline, as fatigue or stress may compromise data quality. Most existing cognitive tasks focus on attention, working memory, or frontal executive functions, which, while valuable, may not fully reflect the cognitive demands encountered in daily life. Simple, intuitive tasks closely related to everyday cognitive processes are therefore needed to diagnose and monitor AD-related cognitive decline in older adults. Additionally, as most early AD diagnosis studies rely on resting-state EEG, comparing task-based data with resting-state data is crucial for obtaining comprehensive insights.

In this study, we aimed to diagnose the AD spectrum using EEG recorded during both resting-state and task-based conditions. Specifically, we focused on a simple yes/no question task designed to mimic everyday cognitive activities. Although the task appears simple, it engages multiple cognitive processes, including listening, comprehension, memory, attention, and response execution. These processes interact dynamically, and impairments in one domain may be compensated for by others, potentially masking deficits in overall cognitive performance. Therefore, we analyzed brain activity during task performance, as it provides critical insights into the cognitive processes underlying AD-related deficits and offers valuable information for early diagnosis. To our knowledge, this is the first study to classify the AD spectrum using EEG recorded during an everyday cognitive task. Additionally, we used raw EEG signals without feature extraction, minimizing human intervention by leveraging state-of-the-art DL models. The objectives of this study were as follows: First, we proposed a task that is simpler, shorter, and more practical than traditional cognitive tasks (e.g., N-back and Go/No-go tasks), making it suitable for older patients with cognitive decline. Second, we compared task-based EEG data with resting-state EEG data, both of which have been commonly used in previous studies. Lastly, we applied advanced artificial intelligence models instead of traditional machine learning techniques to improve classification accuracy. To summarize, the key contributions of this study to the existing literature are as follows.

- Conducted a comprehensive comparison of EEG data recorded during both resting-state and task-based conditions to provide a holistic understanding of brain activity across the AD spectrum;
- Proposed a simple, intuitive, and practical cognitive task (yes/no question task) that mimics everyday cognitive activities, making it more suitable for older adults with cognitive decline compared to traditional cognitive tasks (e.g., N-back or Go/No-go tasks);
- Utilized raw EEG signals without feature extraction, minimizing human intervention, and applied state-of-the-art deep learning models to improve classification performance and generalizability. The study also emphasized reliability by reporting results based on a separate test set rather than relying solely on validation results;
- Focused on assessing the severity within the AD spectrum (SCD, MCI, and AD) rather than simply comparing groups with and without the disease, addressing a critical gap in the literature.

## 2. Materials and Methods

### 2.1. Participants

Sixty participants were recruited from Korea University College of Medicine between 2021 and 2022. The participants were divided into three groups: 20 with SCD, 30 with MCI, and 10 with AD. Two participants were excluded due to missing data or dropout, resulting in a final dataset of 20, 28, and 10 participants with SCD, MCI, and AD, respectively. Table 1 summarizes the demographic and clinical characteristics of the participants, including group distribution, sex, age, years of education, and cognitive test scores.

To assess overall neurocognitive function, the Mini-Mental State Examination (MMSE) was administered to all participants. Additionally, neuropsychological testing using the Seoul Neuropsychological Screening Test-II (SNSB-II) [24] was conducted with patients and/or caregivers who reported subjective memory impairment. Clinical Dementia Rating (CDR) scores were used to classify participants into diagnostic groups: CDR 0 for SCD, CDR 0.5 for MCI, and CDR 1 for AD [25,26]. Detailed cognitive test scores for each group are presented in Table 1.

This study adhered to the ethical principles outlined in the Declaration of Helsinki and was approved by the Institutional Review Board of Korea University Anam Hospital (approval no. 2021AN0266). Written informed consent was obtained from all participants before their inclusion in the study.

### 2.2. Neuropsychological Test

The SNSB-II is a comprehensive neuropsychological test that assesses the following five cognitive domains: (1) Memory: Seoul Verbal Learning Test delayed recall (verbal memory) and Rey–Osterrieth Complex Figure Test (RCFT) delayed recall (visual memory); (2) Language: Korean version of the Boston Naming Test, reading, writing, comprehension, repetition, calculation, finger naming, body part identification, left–right orientation, and praxis tests; (3) Visuospatial Function: Clock Drawing Test and RCFT Copy Test; (4) Frontal Executive Function: motor intolerance, square and triangle crossing, Luria loop, contrast programming, Go/No-go, Fist-Blade-Hand Test, hand movement crossing, digit symbol coding, Korean Trail Making Test, Animal Name and Supermarket Item Task, phoneme portion of the Controlled Oral Word Association Test, and Stroop Test (color reading); (5) Attention: Digit Spacing Antagonism Test, Boundary Test, and Letter Cancellation Test. In cognitive domain scores (Table 2), no significant difference was observed among SCD, MCI, and AD groups in the Attention domain. However, significant differences were identified between SCD and AD in the Language domain, as well as between SCD and MCI and between SCD and AD in the Visuospatial Function, Memory, and Frontal Executive Function domains.

### 2.3. Simple Question Task

In our previous study, we developed a computer-based cognitive assessment consisting of 11 tasks designed to evaluate the five cognitive domains assessed in traditional neuropsychological tests. A detailed description of the full test has been provided previously [27]. Among these tasks, the Simple Question Task (SQT) was specifically designed to replicate the “Comprehension” task in the Language domain of the SNSB-II.

**Figure 1:**
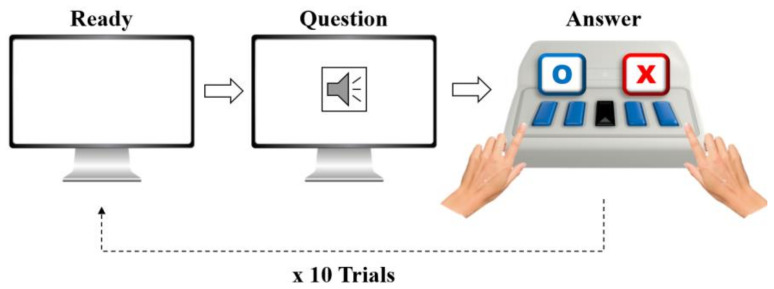
Experimental protocol. Diagram illustrating the process for the Simple Question Task (SQT). Participants completed 10 trials, starting with a “Ready” screen, followed by an auditory “Question” prompt, and responded by pressing the “O” (yes) or “X” (no) buttons. The task was designed to evaluate cognitive responses under controlled conditions.

As shown in Figure 1, the SQT consists of 10 questions that assess everyday semantic knowledge. Participants listen to each question and respond by pressing either the “O” (yes) or “X” (no) button. The duration of each question was kept under 2.2 s to ensure brevity, with participants instructed to respond within 10 s. The list of questions used is provided in Table 3.

This task was designed to replicate everyday cognitive processes, such as listening, understanding, and responding, while maintaining simplicity and accessibility. By focusing on a task that is both familiar and straightforward, the SQT minimizes cognitive load, making it particularly suitable for older individuals and those with cognitive impairments. Although answering questions in the SQT may appear simple, it activates multiple cognitive domains within a short timeframe. For instance, to respond to the question, “Are we in a hospital right now?”, participants engage in complex cognitive processes, including auditory processing, attention, language comprehension, memory retrieval, and executive function. Specifically, participants must process the auditory input (hearing the question), focus on its content, comprehend the sentence structure, retrieve contextual or episodic memory to determine their current location, and execute a motor response by pressing the correct button (O/X). These processes occur in sequence, requiring coordinated activation of multiple brain regions.

Importantly, while the final response (e.g., “yes”) may be identical across participants, underlying brain activity can differ significantly depending on cognitive integrity.

For example, a participant with intact cognitive function will exhibit normal activation across all domains, whereas a participant with impaired attention might still arrive at the correct response by compensating with other cognitive domains, such as language or memory.

This distinction underscores the importance of analyzing brain activity during task performance rather than relying solely on behavioral outcomes. By examining neural patterns associated with task execution, subtle cognitive impairments that may not be evident from behavioral responses alone can be detected. This approach highlights the SQT’s potential as a diagnostic tool for identifying early cognitive changes within the AD spectrum.

### 2.4. Data Acquisition and Preprocessing

EEG signals were acquired during both resting and cognitive task conditions using the ActiveTwo BioSemi system (BioSemi, Amsterdam, The Netherlands). Data were recorded from 64 scalp electrodes positioned according to the 10/20 system, with the CMS/DRL electrode serving as the ground electrode, as specified by BioSemi’s design. EEG signals were digitized at a 2048 Hz sampling rate using ActiView software 8.11 (BioSemi), with impedance maintained below 10 kΩ. EEG data were processed using MATLAB R2022b (MathWorks Inc., Natick, MA, USA) in conjunction with the EEGLAB v2022.1 toolbox. The data were downsampled to 512 Hz and bandpass-filtered with cutoff frequencies of 2–100 Hz. A notch filter at 60 Hz was applied to eliminate electrical line noise. An average reference was used, and Independent Component Analysis (ICA) was applied automatically to remove artifacts such as eye blinks, muscle movements, and electrocardiographic interference. For the SQT data, trials were segmented to include the 1.5 s interval preceding the end of each question. Resting-state data were recorded for 1 min with participants’ eyes closed, and the middle portion of the data was segmented into 1.5 s intervals, resulting in 10 trials to match the number of trials in the SQT data. To streamline the experiment for potential clinical applications, analysis was limited to 19 EEG channels based on channel selection criteria from a previous study [9]. These channels included Fp1, Fp2, F7, F3, Fz, F4, F8, T3, C3, Cz, C4, T4, T5, P3, Pz, P4, T6, O1, and O2.

### 2.5. Classification

EEG time-series data are recorded from multiple scalp locations, capturing two key types of information: temporal activity and spatial relationships between brain signals at different scalp regions. Temporal activity refers to the dynamic changes in brain signals over time, recorded at each channel corresponding to specific brain regions. To model these temporal dependencies, LSTM networks are commonly employed. LSTM, a type of recurrent neural network, is designed to address the vanishing gradient problem and capture long-term dependencies in sequential data. However, LSTM processes information sequentially and relies on the last hidden state, which limits its ability to focus on critical time points. To address this limitation, we incorporated an attention mechanism into the LSTM framework [28,29,30], allowing the model to effectively learn complex patterns by emphasizing important temporal features.

The second type of information, spatial relationships, captures the interactions between brain signals recorded from different scalp locations at specific time points. These spatial features are typically extracted using CNNs. Among the various CNN-based architectures, the EEGConformer [31], which integrates the strengths of both Transformers and CNNs, has been proposed as a state-of-the-art model. This architecture is designed to extract both spatial and temporal features from EEG signals, making it particularly effective for analyzing time-series data.

**Figure 2:**
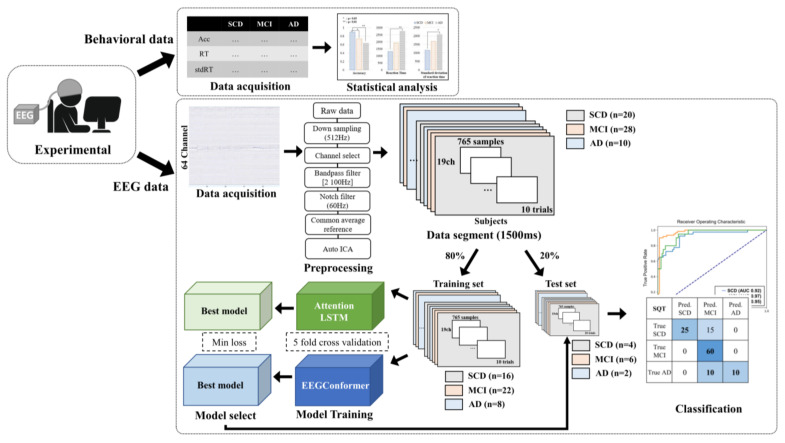
Schematic diagram of the workflow. This figure illustrates the complete workflow of the study, beginning with data acquisition, followed by preprocessing, model training, and evaluation. Behavioral and EEG data were collected, segmented, and divided into training and test sets. Two deep learning models, Attention-LSTM and EEGConformer, were trained and evaluated for classification across the Alzheimer’s disease spectrum (SCD, MCI, AD). Statistical analysis and classification results are displayed, showcasing model performance and ROC curves.

To address the clinical challenge of identifying the AD spectrum, we compared the classification performance of two models: (1) an Attention-LSTM model, which focuses on temporal features, and (2) an EEGConformer model, which integrates both spatial and temporal features. By using minimally preprocessed EEG data, we aimed to automatically extract hidden patterns, including features that are difficult for humans to detect. Additionally, we conducted an analysis using an SVM implemented with the sklearn library in Python to compare with traditional models. Principal Component Analysis (PCA) was applied to reduce the dimensionality of the data while retaining 95% of the total variance. The number of principal components was automatically determined based on the explained variance ratio threshold of 0.95. This comparison allowed us to evaluate the relative strengths of temporal-focused and spatial–temporal hybrid approaches in classifying the AD spectrum. We implemented a pipeline to classify EEG epochs, as illustrated in Figure 2, by designing and training both the Attention-LSTM model and EEGConformer on SQT signals from participants with SCD, MCI, and AD. The same pipeline was applied to classify SCD, MCI, and AD using resting-state EEG signals.

#### 2.5.1. Attention-LSTM

**Figure 3:**
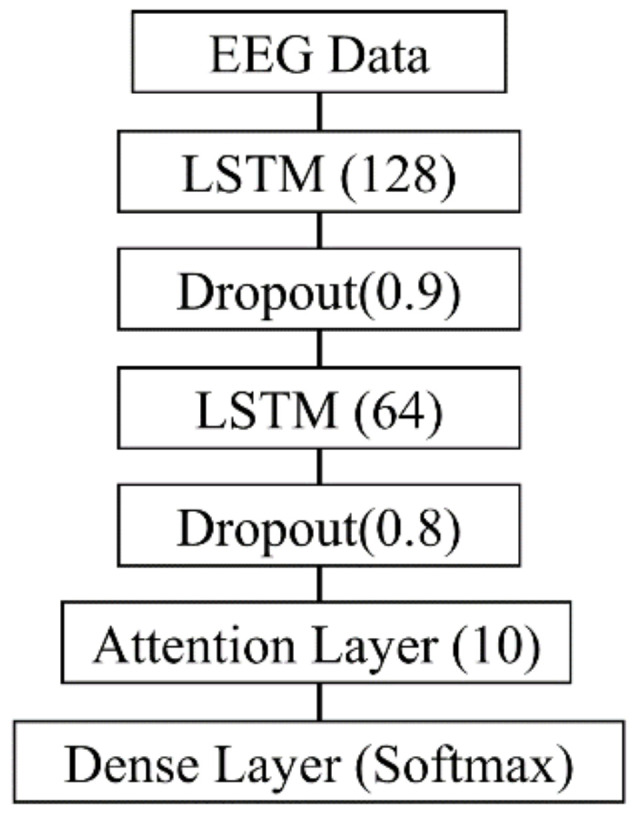
The framework of Attention-LSTM. This figure depicts the architecture of the Attention-LSTM model used in the study. The model processes EEG data through two LSTM layers (128 and 64 units), each followed by a dropout layer (rates 0.9 and 0.8). An attention layer with 10 units highlights key temporal features, and a dense layer with a softmax activation function performs the final classification.

The Attention-LSTM model was implemented and trained, as illustrated in Figure 3, using Python 3.10.13 with the Keras library. Evaluation algorithms were implemented using scikit-learn. The training was conducted on an NVIDIA RTX 3070 Ti GPU with CUDA 11.2. The input data consisted of an EEG time series with a shape of 768 samples × 19 channels.

The model architecture included the following components: The first LSTM layer, consisting of 128 units, was designed to capture temporal dependencies in the EEG data and pass the full sequence to the next layer. The second LSTM layer, consisting of 64 units, refined the temporal patterns learned by the first layer. Dropout layers were applied after each LSTM layer to prevent overfitting and improve generalization, with dropout rates set to 0.9 and 0.8, respectively. The attention layer was applied to the output of the LSTM layers to assign weights to important time points in the sequence. This layer used 10 units to compute attention scores for each element, generating a context vector that enabled the model to focus on critical information. The dense output layer utilized a softmax activation function for multi-class classification.

The model was trained using the Adam optimizer with a learning rate of 0.001 and a categorical cross-entropy loss function appropriate for multi-class classification tasks with one-hot encoded labels. Training was conducted for up to 1000 epochs with a batch size of 64. Class weights were applied using scikit-learn to address the class imbalance and ensure balanced performance across all classes.

To prevent overfitting, an early stopping mechanism was implemented to monitor the validation loss. Training was halted if no improvement was observed over 10 consecutive epochs, with model weights restored to the best-performing configuration. The minimum improvement threshold was set to 0, ensuring that even minor improvements were captured.

This training and evaluation strategy enabled the Attention-LSTM model to achieve robust generalization performance while minimizing overfitting.

#### 2.5.2. EEGConformer

The EEGConformer model, originally proposed by Song et al. [31], was adapted to fit our dataset by modifying parameters such as the number of channels. Training was conducted on an NVIDIA RTX 3070 Ti GPU with CUDA 11.2 using a batch size of 100 and a learning rate of 0.001. As with the Attention-LSTM model, class weights were applied to address class imbalance, and training was conducted for up to 1000 epochs with early stopping.

In addition to early stopping, the EEGConformer model incorporated data augmentation to improve generalization and prevent overfitting. The number of augmented samples was limited to 20% of the original dataset to maintain a balance between diversity and reliability. Furthermore, we employed ReduceLROnPlateau, a PyTorch method that dynamically adjusts the learning rate based on changes in the loss function, optimizing the learning process and ensuring stable convergence.

The EEGConformer architecture integrates the strengths of CNNs and Transformers, enabling it to capture both spatial and temporal features of EEG signals. This hybrid approach is particularly effective for analyzing complex time-series data, such as EEG.

#### 2.5.3. Model Evaluation

To ensure the reliability of our results, we adopted a rigorous evaluation strategy for the models. The dataset was divided into two parts: 80% for training and validation and 20% for testing. The test set consisted exclusively of data that were not used during the training process, ensuring an unbiased evaluation of the model’s performance. During training, 5-fold cross-validation was applied to optimize the model. The model that performed best on the validation set was selected for final testing on the independent test set.

As reported previously [21], EEG data are inherently subject-specific, meaning that even trials from different time segments of the same subject can share unique patterns. If trials from the same subject are mixed between the training and test sets (trial-wise splitting), the model may overfit to subject-specific features rather than generalize to unseen data. To mitigate this issue, we adopted a subject-wise splitting approach, ensuring that trials from the same subject were not shared between the training and test sets. This method prevents overfitting and enhances the model’s generalizability.

To comprehensively evaluate the models’ performance, we used several metrics, including accuracy (Equation (1)), recall (Equation (2)), precision (Equation (3)), F1 score (Equation (4)), and the area under the receiver operating characteristic curve (AUC). These metrics were calculated based on true positives (*TP*), true negatives (*TN*), false positives (*FP*), and false negatives (*FN*). Given the class imbalance in our dataset, the F1 score was weighted according to the number of samples in each class, ensuring a balanced assessment of the model’s performance. This multi-faceted evaluation framework ensured that the reported results were robust, reliable, and reflective of the model’s ability to classify the AD spectrum effectively.

(1) $$Accuracy=\frac{TP+TN}{TP+TN+FP+FN}$$

(2) $$Recall=\frac{TP}{TP+FN}$$

(3) $$Precision=\frac{TP}{TP+FP}$$

(4) $$F1\;score=2\times \frac{Precision\times Recall}{Precision+Recall}$$

### 2.6. Statistical Analysis

To evaluate statistical differences between the groups, we assessed the normality of the data using the Shapiro–Wilk test, which revealed that the data did not follow a normal distribution. Consequently, the nonparametric Kruskal–Wallis test was employed to compare the groups, followed by post hoc pairwise comparisons to identify specific group differences. All statistical analyses were conducted using SPSS version 22.0.

## 3. Results

### 3.1. Behavioral Results

In the traditional neuropsychological “Comprehension Test”, all participants from the SCD, MCI, and AD groups answered all five questions correctly, making it impossible to differentiate between the groups based on accuracy alone. To address this limitation, the proposed SQT increased the number of questions to 10 and incorporated both accuracy and reaction time (RT) as additional measures to distinguish between the AD spectrum To evaluate whether the SQT was sufficiently challenging for differentiating the SCD, MCI, and AD groups, we analyzed accuracy, RT, and the standard deviation of reaction times (stdRT) across the 10 trials for each participant. The results are summarized in Table 4.

**Figure 4:**
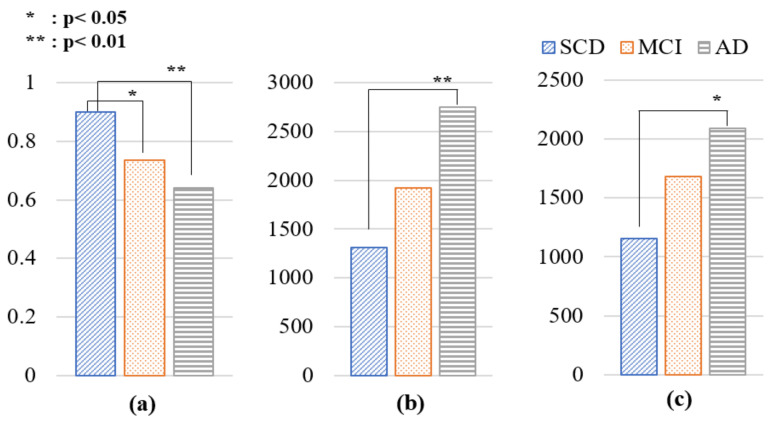
Comparison of SQT behavioral results by group. (**a**) Accuracy, (**b**) reaction time, (**c**) standard deviation of reaction time. SQT: Simple Question Task; SCD: subjective cognitive decline; MCI: mild cognitive impairment; AD: Alzheimer’s disease.

As shown in Figure 4, the SCD group achieved an average accuracy of 90%, while the MCI and AD groups achieved accuracies of 73.5% and 64.0%, respectively. These findings indicate that participants in the AD group experienced greater difficulty completing the task compared to those in the SCD and MCI groups. Significant differences in accuracy were observed between the SCD and MCI groups (*p* = 0.018) and between the SCD and AD groups (*p* = 0.005).

Regarding RT, the SCD group had an average RT of 1312.816 ms, while the MCI and AD groups had average RTs of 1924.975 ms and 2751.7 ms, respectively. These results demonstrate that participants with more severe cognitive impairment took significantly longer to respond to the questions. Additionally, stdRT across the 10 trials was significantly higher in the MCI (1683.482 ms) and AD (2090.829 ms) groups than in the SCD group (1155.313 ms), suggesting greater variability in response times among individuals with cognitive impairment. Significant differences in RT (*p* = 0.002) and stdRT (*p* = 0.022) were observed between the SCD and AD groups.

These findings suggest that the behavioral outcomes of the SQT effectively differentiate between the SCD and MCI/AD groups. Accuracy, RT, and variability in reaction time serve as meaningful behavioral indicators of cognitive impairment.

### 3.2. Classification Results

The analysis confirmed that the SVM model failed to classify the data properly and that there was overfitting to the SCD group. This outcome is likely due to the inherent limitations of traditional pattern recognition methods, which rely on hand-crafted feature selection from raw data before classification. In contrast, this study utilized deep learning models trained on automatically extracted features from raw EEG, which may not align well with the feature requirements of the SVM model, leading to suboptimal results. In contrast, the deep learning-based models, EEGConformer and Attention-LSTM, trained effectively and demonstrated robust classification performance, with no significant difference observed between the two models. To evaluate the effectiveness of the EEG state (resting-state- versus cognitive-task-based) in classifying the AD spectrum, we compared the classification performance of EEG data collected during the resting state (REST) and the SQT. The results are summarized in Table 5, Table 6 and Table 7.

For distinguishing between SCD and MCI, REST data achieved slightly higher classification accuracy (92% for EEGConformer and 88% for Attention-LSTM) compared to SQT data (87% for EEGConformer and 85% for Attention-LSTM). However, for distinguishing between MCI and AD, SQT data outperformed REST data, with classification accuracies of 88.75% (SQT) versus 73.75–78.75% (REST), depending on the model. Similarly, for classifying all three groups (SCD, MCI, and AD), SQT data yielded higher accuracies (76.66–79.16%) compared to REST data (71.67%).

**Figure 5:**
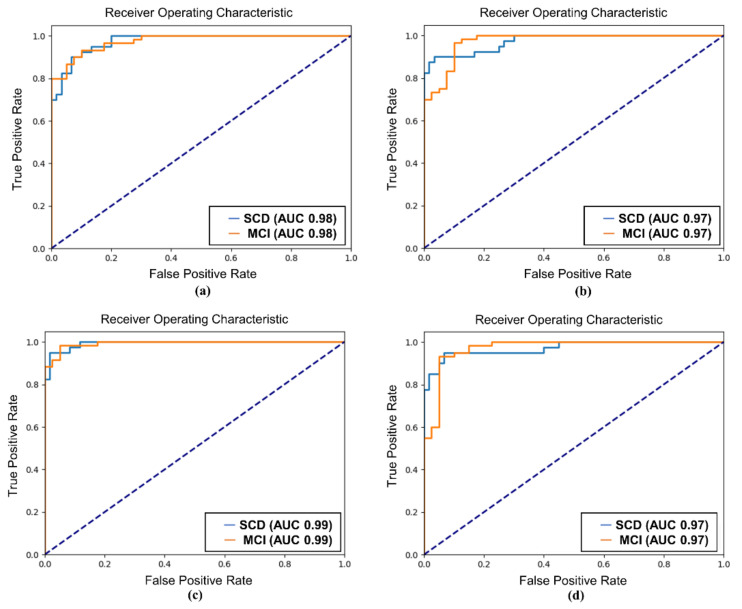
ROC curve of SCD and MCI classification. (**a**) EEGConformer-SQT; (**b**) EEGConformer-REST; (**c**) Attention-LSTM-SQT; (**d**) Attention-LSTM-REST; REST: EEG data collected during the resting state; SQT: Simple Question Task; ROC: receiver operating characteristic; LSTM: long short-term memory; SCD: subjective cognitive decline; MCI: mild cognitive impairment.

**Figure 6:**
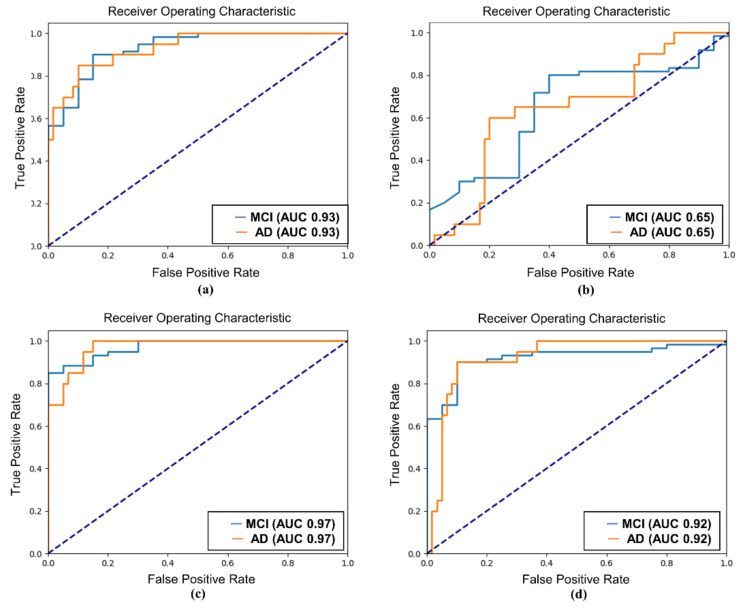
ROC curve of MCI and AD classification. (**a**) EEGConformer-SQT; (**b**) EEGConformer-REST; (**c**) Attention-LSTM-SQT; (**d**) Attention-LSTM-RESTREST: EEG data collected during the resting state; SQT: Simple Question Task; ROC: receiver operating characteristic; LSTM: long short-term memory; MCI: mild cognitive impairment; AD: Alzheimer’s disease.

**Figure 7:**
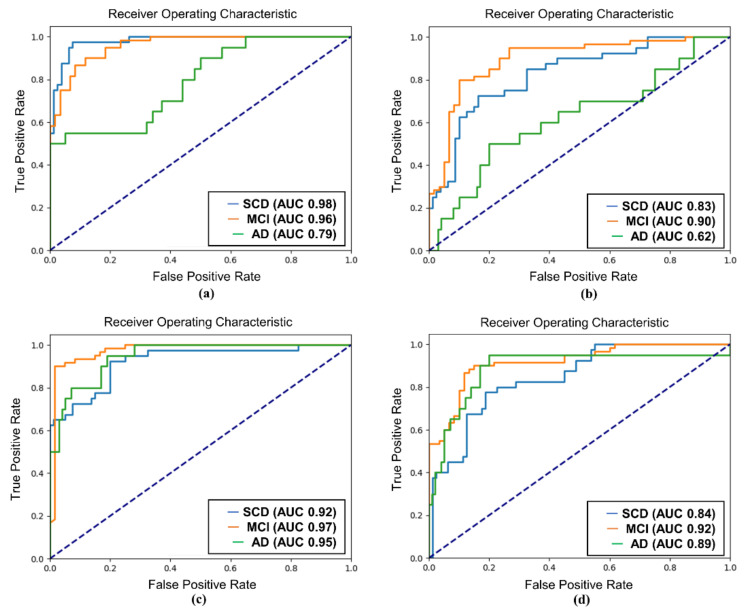
ROC curve of SCD, MCI, and AD classification. (**a**) EEGConformer-SQT; (**b**) EEGConformer-REST; (**c**) Attention-LSTM-SQT; (**d**) Attention-LSTM-REST; REST: EEG data collected during the resting state; SQT: Simple Question Task; ROC: receiver operating characteristic; LSTM: long short-term memory; SCD: subjective cognitive decline; MCI: mild cognitive impairment; AD: Alzheimer’s disease.

The AUC values further supported the superiority of SQT data for classification. For SCD and MCI classification, the SQT data achieved AUC values of 0.99 (Attention-LSTM) and 0.98 (EEGConformer), compared to slightly lower AUC values of 0.97 for the REST data. For MCI and AD classification, the SQT data again outperformed the REST data, with AUC values of 0.97 (Attention-LSTM) and 0.93 (EEGConformer) for SQT, compared to lower AUC values of 0.92 (Attention-LSTM) and 0.65 (EEGConformer) for REST. When classifying all three groups (SCD, MCI, and AD), the SQT data yielded high AUC values for both models. The Attention-LSTM model reported AUC values of 0.92 for SCD, 0.97 for MCI, and 0.95 for AD, while the EEGConformer reported AUC values of 0.98 for SCD, 0.96 for MCI, and 0.79 for AD (Figure 5, Figure 6 and Figure 7).

The confusion matrix results provided additional insights into the classification performance of the models. For SCD and MCI classification, the REST data outperformed the SQT data in classifying SCD trials. For instance, with REST data, the EEGConformer model correctly classified 33 of 40 SCD trials, while the Attention-LSTM model correctly classified 29 of 40 trials. In contrast, using SQT data, the EEGConformer model correctly classified 27 of 40 SCD trials, and the Attention-LSTM model classified 25 of 40 trials. However, for MCI trials, both models demonstrated nearly perfect classification performance regardless of the data type.

For MCI and AD classification, the SQT data outperformed the REST data. Using SQT data, the EEGConformer model correctly classified 59 of 60 MCI trials and 12 of 20 AD trials, while the Attention-LSTM model correctly classified 60 of 60 MCI trials and 11 of 20 AD trials. Conversely, using REST data, the EEGConformer model correctly classified only 48 of 60 MCI trials and 11 of 20 AD trials, while the Attention-LSTM model correctly classified 59 of 60 MCI trials and only four of 20 AD trials.

**Figure 8:**
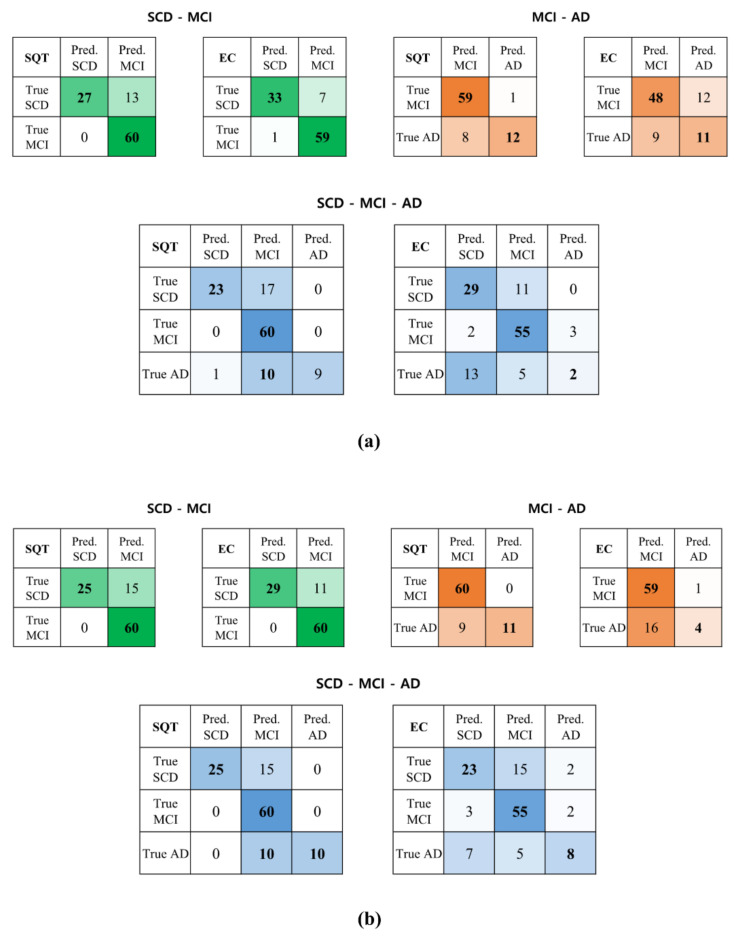
Confusion matrix for SCD, MCI, and AD. (**a**) EEGConformer; (**b**) Attention-LSTM. REST: EEG data collected during the resting state; SQT: Simple Question Task; LSTM: long short-term memory; SCD: subjective cognitive decline; MCI: mild cognitive impairment; AD: Alzheimer’s disease.

When classifying all three groups(SCD, MCI, and AD), MCI was consistently the easiest group to classify, followed by SCD and AD. For SCD trials, REST data outperformed SQT data, with fewer misclassifications. For example, with REST data, the EEGConformer model misclassified 11 SCD trials as MCI, while the Attention-LSTM model misclassified 15 SCD trials as MCI. However, for AD trials, the SQT data outperformed the REST data. Using SQT data, the EEGConformer model misclassified 10 AD trials as MCI and one AD trial as SCD, while the Attention-LSTM model misclassified 10 AD trials as MCI and none as SCD. In contrast, with REST data, the EEGConformer model misclassified five AD trials as MCI and 13 AD trials as SCD, while the Attention-LSTM model misclassified five AD trials as MCI and seven AD trials as SCD (Figure 8).

At the participant level, classification accuracy was determined by whether more than 50% of a participant’s 10 trials were correctly classified [9]. Using SQT data, the EEGConformer model correctly classified three of four participants with SCD, all six participants with MCI, and one of two participants with AD. In contrast, with REST data, the EEGConformer model correctly classified all four participants with SCD and all six participants with MCI but misclassified both participants with AD as having SCD.

For the Attention-LSTM model, using SQT data, two of four participants with SCD were misclassified as having MCI, while all six participants with MCI were correctly classified, and one of two participants with AD was misclassified as having MCI. With REST data, two of four participants with SCD were misclassified as having MCI, all six participants with MCI were correctly classified, and one of two participants with AD was misclassified as having SCD. These results are detailed in Table 8 and Table 9.

Overall, the results demonstrate that the SQT data offer superior performance for classifying AD spectrum groups, particularly for distinguishing MCI and AD, while REST data perform slightly better for classifying SCD. The EEGConformer model generally outperformed the Attention-LSTM model for SCD classification, whereas the Attention-LSTM model showed better performance for AD classification.

## 4. Discussion

In this study, we introduced the SQT, a modified version of the traditional “Comprehension” neuropsychological task, designed to engage relatively simple yet cognitively complex processes. By applying DL techniques to EEG data recorded during the SQT, we aimed to identify patterns of cognitive processing that could be used to classify individuals across the AD spectrum. Unlike conventional task-based EEG studies, which typically require hundreds of trials and testing durations exceeding 30 min (as shown in Table 10 and Table 11), our approach significantly reduces testing time to under 3 min. The SQT consists of 10 straightforward questions, each lasting 1–2 s. This reduction in task duration offers several advantages in clinical settings. Shorter testing times are particularly beneficial for older individuals and patients with AD, who often have limited attention spans and are more prone to data contamination caused by fatigue or cognitive overload. By minimizing these risks, the brevity of the SQT enhances its feasibility for real-world medical applications, making it a practical and efficient tool for rapid cognitive assessment.

In traditional neuropsychological tests, such as paper-and-pencil-based comprehension tasks, all groups, including patients with SCD, MCI, and AD, answered all five questions correctly, making it difficult to distinguish between groups. In contrast, the behavioral results of the SQT revealed significant differences in accuracy between the SCD and MCI groups, as well as between the SCD and AD groups. No significant differences in accuracy were observed between the MCI and AD groups. Similarly, RTs progressively increased from SCD to AD, and variability in RTs across trials also increased. Significant differences in RTs were observed only between the SCD and AD groups. Neither accuracy nor RT showed significant differences between the MCI and AD groups. Despite these limitations in behavioral data, the classification performance based on brain activity during the SQT yielded excellent results. Specifically, the DL model achieved classification accuracies of 87% for SCD vs. MCI, 88.75% for MCI vs. AD, and 79.16% for three-class classification (SCD, MCI, and AD). Notably, the model successfully distinguished between MCI and AD, which behavioral data alone could not accomplish. These results surpass the classification performance reported in previous studies (Table 10), underscoring the robustness of the proposed approach. Furthermore, the model was validated on a subject-wise basis, reinforcing the reliability and generalizability of the findings.

When comparing SQT-based EEG data with resting-state EEG data, classification accuracy for SCD vs. MCI was higher with resting-state EEG. However, the AUC, a key metric for evaluating classification performance, was higher for SQT-based EEG. AUC is particularly valuable for datasets with class imbalances, demonstrating the utility of SQT-based EEG in such scenarios. Additionally, for both binary classification (MCI vs. AD) and three-class classification (SCD, MCI, AD), SQT-based EEG consistently outperformed resting-state EEG, with accuracy improvements ranging from 5% to 15%. These results highlight that EEG signals recorded during cognitive tasks provide additional discriminatory power for AD spectrum classification compared to resting-state EEG. This finding underscores the importance of incorporating brain activity during cognitive processes into AD spectrum diagnosis, complementing existing resting-state approaches.

Another significant contribution of this study is the minimal preprocessing required for the proposed AD spectrum classification model. By reducing the need for manual intervention, the model moves closer to achieving an automated AD diagnostic system. We compared two state-of-the-art DL models: the Attention-LSTM model, which leverages the temporal characteristics of the data, and the EEGConformer model, which accounts for both temporal and spatial features. Neither model consistently outperformed the other, suggesting that the quality and characteristics of the input data are more critical than the specific model architecture.

A limitation of this study is that the DL method we applied faces challenges in providing neurophysiological interpretation of the results because the model automatically extracts and learns features without explicitly identifying biomarkers. Further research on the neurophysiological basis of EEG biomarkers during cognitive tasks is necessary across various fields. Additionally, the unequal proportions of each group and the small sample size represent limitations of the study, implying difficulties in ensuring generalizability. In future studies, we plan to collect more data to increase the reliability of the results.

In conclusion, the proposed SQT-based EEG framework demonstrates significant potential for AD spectrum classification, offering a fast, practical, and effective alternative to traditional neuropsychological tests and resting-state EEG approaches. This study paves the way for more accurate and efficient diagnostic tools for AD spectrum disorders by utilizing brain activity during cognitive processes. Future research should prioritize expanding biomarker identification and integrating neurophysiological insights to address the interpretation challenges of DL models in this domain.

## Figures and Tables

**Table 1:** Demographic and clinical characteristics.

Group	SCD	MCI	AD	adj. *p* Values	
N	20	28	10		
Sex (F:M)	9:11	15:13	6:4		
Age in years (SD)	63.39 (11.19)	67.39 (7.63)	71.20 (7.13)	0.074	
Years of education (SD)	8.43 (5.14)	10.04 (4.55)	10.00 (4.22)	0.753	
MMSE (SD)	26.95 (6.54)	26.14 (2.88)	23.40 (3.10)	**1.627 × 10^−4^**	**SCD-MCI: 0.024** MCI-AD: 0.086 **SCD-AD: 1.354 × 10^−4^**
SGDS	4.30	6.00	6.40	0.437	
B-ADL	19.00	19.39	17.50	0.096	
K-IADL	0.05	0.20	0.45	**3.169 × 10^−6^**	**SCD-MCI: 0.006** **MCI-AD: 0.015** **SCD-AD: 2.533 × 10^−6^**
CDR	0.20	0.48	0.60	**5.568 × 10^−6^**	**SCD-MCI: 3.676 × 10^-4^** MCI-AD: 0.260 **SCD-AD: 2.648 × 10^−5^**
GDS	1.90	2.93	3.50	**2.070 × 10^−9^**	**SCD-MCI:1.334 × 10^−6^** MCI-AD: 0.152 **SCD-AD: 3.9756 × 10^−8^**

MMSE: Mini-Mental State Examination; SGDS: short version of the Geriatric Depression Scale; B-ADL: Barthel Activities of Daily Living; K-IADL: Korean Instrumental Activities of Daily Living; CDR: Clinical Dementia Rating; GDS: Global Deterioration Scale; SCD: subjective cognitive decline; MCI: mild cognitive impairment; AD: Alzheimer’s disease; bold text indicates *p* < 0.05.

**Table 2:** Cognitive domain scores in the neuropsychological test.

Group	SCD	MCI	AD	adj. *p* Values	
Attention	0.72	0.33	−0.14	0.101	
Language	0.51	0.03	−1.35	**0.002**	SCD-MCI: 0.150 MCI-AD: 0.081 **SCD-AD: 0.001**
Visuospatial Function	0.44	−0.33	−1.65	**1.681 × 10^−4^**	**SCD-MCI: 0.015** MCI-AD: 0.119 **SCD-AD: 1.873 × 10^−4^**
Memory	0.28	−1.43	−2.39	**2.020 × 10^−7^**	**SCD-MCI:2.510 × 10^−5^** MCI-AD: 0.241 **SCD-AD: 2.481 × 10^−6^**
Frontal Executive Function	−0.20	−1.34	−2.66	**2.498 × 10^−5^**	**SCD-MCI: 1.798 × 10^−4^** MCI-AD: 1.000 **SCD-AD: 5.668 × 10^−4^**

SCD: subjective cognitive decline; MCI: mild cognitive impairment; AD: Alzheimer’s disease; bold text indicates *p* < 0.05.

**Table 3:** Question list for the Simple Question Task.

Question List	Correct Answer	Listening Time (ms)
Q1. Is this school now?	X	1533.5
Q2. Is the number 9 greater than 6?	O	1948.75
Q3. Do stones sink in water?	O	1533.25
Q4. Is a hammer used for cutting wood?	X	1942.75
Q5. Do you put on your socks before you put on your shoes?	O	2200
Q6. Is Wednesday the day after Tuesday?	O	1948.5
Q7. Does it snow in May?	X	1380
Q8. Is a cat bigger than a mouse?	O	1795.5
Q9. Are birds eaten by bugs?	X	1792
Q10. Are banks open on Sunday?	X	1791.25

**Table 4:** Behavioral results of SQT.

	SCD	MCI	AD	*p*	
Acc (SD)	90.00% (0.13)	73.50% (0.216)	64.00% (0.237)	0.002	**SCD-MCI: 0.018** MCI-AD: 0.596 **SCD-AD: 0.005**
RT (SD)	1312.816 ms (491.698)	1924.975 ms (942.935)	2751.7 ms (1204.215)	0.002	SCD-MCI: 0.079 MCI-AD: 0.195 **SCD-AD: 0.002**
stdRT (SD)	1155.313 ms (695.829)	1683.482 ms (927.305)	2090.829 ms (862.255)	0.019	SCD-MCI: 0.142 MCI-AD: 0.507 **SCD-AD: 0.022**

Acc: accuracy; RT: reaction time; SD: standard deviation among participants; stdRT: standard deviation of reaction time among trials; SCD: subjective cognitive decline; MCI: mild cognitive impairment; AD: Alzheimer’s disease; bold text indicates *p* < 0.05.

**Table 5:** Results of classifying SCD and MCI using deep learning models.

(Weighted avg.)		Accuracy	Precision	Recall	F1	AUC
SQT	SVM	40.00%	0.16	0.40	0.23	0.54, 0.00
	EEGConformer	87.00%	0.89	0.87	0.86	0.98, 0.98
	Attention-LSTM	85.00%	0.88	0.85	0.85	**0.99, 0.99**
REST	SVM	40.00%	0.16	0.40	0.23	0.61, 0.00
	EEGConformer	**92.00%**	**0.92**	**0.92**	**0.92**	0.97, 0.97
	Attention-LSTM	88.00%	0.91	0.89	0.89	0.97, 0.97

REST: EEG data collected during the resting state; SQT: Simple Question Task; AUC: area under the receiver operating characteristic curve; LSTM: long short-term memory; bold text indicates the best performance.

**Table 6:** Results of classifying MCI and AD using deep learning models.

(Weighted avg.)		Accuracy	Precision	Recall	F1	AUC
SQT	SVM	75.00%	0.56	0.75	0.64	0.59, 0.00
	EEGConformer	**88.75%**	0.89	**0.89**	**0.88**	0.93, 0.93
	Attention-LSTM	**88.75%**	**0.90**	**0.89**	**0.88**	**0.97, 0.97**
REST	SVM	75.00%	0.56	0.75	0.64	0.60, 0.00
	EEGConformer	73.75%	0.75	0.74	0.74	0.65, 0.65
	Attention-LSTM	78.75%	0.79	0.79	0.74	0.92, 0.92

REST: EEG data collected during the resting state; SQT: Simple Question Task; AUC: area under the receiver operating characteristic curve; LSTM: long short-term memory; bold text indicates the best performance.

**Table 7:** Results of classifying SCD, MCI, and AD using deep learning models.

(Weighted avg.)		Accuracy	Precision	Recall	F1	AUC
SQT	SVM	33.33%	0.11	0.33	0.17	0.57, 0.00, 0.00
	EEGConformer	76.66%	0.83	0.77	0.75	0.98, 0.96, 0.79
	Attention-LSTM	**79.16%**	**0.85**	**0.79**	**0.78**	**0.92, 0.97, 0.95**
REST	SVM	33.33%	0.11	0.33	0.17	0.56 0.00, 0.00
	EEGConformer	71.67%	0.67	0.72	0.68	0.83, 0.90, 0.62
	Attention-LSTM	71.67%	0.71	0.72	0.7	0.84, 0.92, 0.89

REST: EEG data collected during the resting state; SQT: Simple Question Task; AUC: area under the receiver operating characteristic curve; LSTM: long short-term memory; bold text indicates the best performance.

**Table 8:** Classification results for individual trials of each participant based on EEGConformer. Bold text indicates the class that was finally classified in more than 50% of the 10 trials, and underlined text indicates misclassified results.

EEGConformer	SQT			REST		
	SCD	MCI	AD	SCD	MCI	AD
**SCD 1**	10%	**90%**	0%	**70%**	30%	0%
**SCD 2**	**80%**	20%	0%	**100%**	0%	0%
**SCD 3**	**90%**	10%	0%	**60%**	40%	0%
**SCD 4**	**50%**	**50%**	0%	**60%**	40%	0%
**MCI 1**	0%	**100%**	0%	0%	**100%**	0%
**MCI 2**	0%	**100%**	0%	0%	**100%**	0%
**MCI 3**	0%	**100%**	0%	0%	**80%**	20%
**MCI 4**	0%	**100%**	0%	0%	**100%**	0%
**MCI 5**	0%	**100%**	0%	0%	**90%**	10%
**MCI 6**	0%	**100%**	0%	20%	**80%**	0%
**AD 1**	0%	**100%**	0%	**50%**	**50%**	0%
**AD 2**	10%	0%	**90%**	**80%**	0%	20%

SCD: subjective cognitive decline; MCI: mild cognitive impairment; AD: Alzheimer’s disease.

**Table 9:** Classification results for individual trials of each participant based on Attention-LSTM. Bold text indicates the class that was finally classified in more than 50% of the 10 trials, and underlined text indicates misclassified results.

Attention-LSTM	SQT			REST		
	SCD	MCI	AD	SCD	MCI	AD
**SCD 1**	30%	**70%**	0%	**70%**	30%	0%
**SCD 2**	**90%**	10%	0%	**100%**	0%	0%
**SCD 3**	**100%**	0%	0%	30%	**70%**	0%
**SCD 4**	30%	**70%**	0%	30%	**50%**	20%
**MCI 1**	0%	**100%**	0%	0%	**100%**	0%
**MCI 2**	0%	**100%**	0%	0%	**100%**	0%
**MCI 3**	0%	**100%**	0%	0%	**90%**	10%
**MCI 4**	0%	**100%**	0%	0%	**100%**	0%
**MCI 5**	0%	**100%**	0%	0%	**90%**	10%
**MCI 6**	0%	**100%**	0%	30%	**70%**	0%
**AD 1**	0%	**100%**	0%	**70%**	30%	0%
**AD 2**	0%	0%	**100%**	0%	20%	**80%**

SCD: subjective cognitive decline; MCI: mild cognitive impairment; AD: Alzheimer’s disease.

**Table 10:** Comparison of results with those of previous studies using resting EEG.

Title	N	Model	Time Taken	Group	Evaluation	Results	
						Acc	AUC
[15], 2023	HCs 29, AD 36	CNN + Transformer	10 min	HCs: AD	LOOCV	83.28%	0.83
[9], 2023	HCs 17, SCD 56, MCI 45	Attention-LSTM	20 min	SCD: MCI	LOOCV	76.20%	0.8
				HCs: SCD: MCI		54.20%	0.74
[18], 2022	HCs 16, MCI 11	LSTM	30 min	HCs: MCI	5-CV	96.41%	0.96
[32], 2023	HCs 459, MCI 417, AD 311	CEEDNet	13 min	HCs + MCI: AD	Test	79.16%	0.83, 0.83
				HCs: MCI: AD		74.66%	0.95, 0.81, 0.92
[19], 2023	86 AD + MCI, 23 HCs	EWGLO-OTA-LSTM	5 min	HCs: MCI + AD	Test	96.17%	-
[20], 2023	HCs 24, AD 24	LSTM + CNN	-	HCs: AD	Test	93.00%	0.97
Ours, 2025	SCD 20, MCI 28, AD 10	EEGConformer/Attention-LSTM	1 min	SCD: MCI	Test	92.00%	0.97, 0.97
				MCI: AD		78.75%	0.92, 0.92
				SCD: MCI: AD		71.67%	0.84, 0.92, 0.89

HC: health controls; SCD: subjective cognitive decline; MCI: mild cognitive impairment; AD: Alzheimer’s disease; EWGLO-OTA-LSTM: Enhanced Wild Geese Lemurs Optimizer-Optimized Transformer-based Attention-LSTM; LOOCV: leave-one-out cross validation; 5-CV: 5-fold cross validation; Acc: accuracy; AUC: area under the receiver operating characteristic curve.

**Table 11:** Comparison of results with those of previous studies using task EEG.

Title	N	Model	Task	Group	Evaluation	Results	
						Acc	AUC
[22], 2021	HCs 23, AD 40	CNN + LSTM	N-back test	HCs: AD	-	73.70%	-
	HCs 13, AD 23		Oddball test			75.95%	-
[23], 2023	YA 30, OA 39, MCI 27	SNN	1-back word test (300 trials)	Young adult: older adult: MCI	10-CV	73.94%	-
			1-back PW test (600 trials)			83.33%	-
			1-back CW test (600 trials)			80.00%	-
Ours, 2025	SCD 20, MCI 28, AD 10	EEGConformer/Attention-LSTM	SQT (10 trials)	SCD: MCI	Test	87.00%	0.98, 0.98
				MCI: AD		88.75%	0.97, 0.97
				SCD: MCI: AD		79.16%	0.92, 0.97, 0.95

HC: health controls; SCD: subjective cognitive decline; MCI: mild cognitive impairment; AD: Alzheimer’s disease; YA: young adult; OA: older adult; PW: perceptual word; CW: conceptual word; SQT: Simple Question Task; 10-CV: 10-fold cross validation; Acc: accuracy; AUC: area under the receiver operating characteristic curve.

## Data Availability

The datasets presented in this article are not readily available because they are part of an ongoing study. Requests to access the datasets should be directed to seulki120@kist.re.kr.

## Abbreviations

The following abbreviations are used in this manuscript:

AD	Alzheimer’s disease
SCD	subjective cognitive decline
MCI	mild cognitive impairment
EEG	electroencephalography
DL	deep learning
CNN	convolutional neural networks
LSTM	long short-term memory
AUC	area under the curve
ROC	receiver operating characteristic
EEGConformer	EEGConformer model
RT	reaction time
MMSE	Mini-Mental State Examination
SNSB-II	Seoul Neuropsychological Screening Test-II
CDR	Clinical Dementia Rating
GDS	Global Deterioration Scale
K-IADL	Korean Instrumental Activities of Daily Living
SGDS	short version of the Geriatric Depression Scale
B-ADL	Barthel Activities of Daily Living
